# An experimental test of CSR theory using a globally calibrated ordination method

**DOI:** 10.1371/journal.pone.0175404

**Published:** 2017-04-07

**Authors:** Yuanzhi Li, Bill Shipley

**Affiliations:** Département de Biologie, Université de Sherbrooke, Sherbrooke, Québec, Canada; Chinese Academy of Forestry, CHINA

## Abstract

Can CSR theory, in conjunction with a recently proposed globally calibrated CSR ordination (“StrateFy”), using only three easily measured leaf traits (leaf area, specific leaf area and leaf dry matter content) predict the functional signature of herbaceous vegetation along experimentally manipulated gradients of soil fertility and disturbance? To determine this, we grew 37 herbaceous species in mixture for five years in 24 experimental mesocosms differing in factorial levels of soil resources (stress) and density-independent mortality (disturbance). We measured 16 different functional traits and then ordinated the resulting vegetation within the CSR triangle using StrateFy. We then calculated community-weighted mean (CWM) values of the competitor (*C*_CWM_), stress-tolerator (*S*_CWM_) and ruderal (*R*_CWM_) scores for each mesocosm. We found a significant increase in *S*_CWM_ from low to high stress mesocosms, and an increase in *R*_CWM_ from lowly to highly disturbed mesocosms. However, *C*_CWM_ did not decline significantly as intensity of stress or disturbance increased, as predicted by CSR theory. This last result likely arose because our herbaceous species were relatively poor competitors in global comparisons and thus no strong competitors in our species pool were selectively favoured in low stress and low disturbed mesocosms. Variation in the 13 other traits, not used by StrateFy, largely argeed with the predictions of CSR theory. StrateFy worked surprisingly well in our experimental study except for the *C*-dimension. Despite loss of some precision, it has great potential applicability in future studies due to its simplicity and generality.

## Introduction

Grime’s CSR model of plant strategies [1–3] has been proposed as a framework for both functionally classifying plants and for predicting how plant community structure changes along environmental gradients. It is perhaps the most influential modern niche-based theory of plant community assembly, vegetation succession and ecosystem functioning and continues to heavily influence the field as shown by current citation rates. CSR theory assumes that variation in the functional response of plants can be predicted and explained by differences in the intensity of stress and disturbance in a local site. Stress [2] is defined as all “external constraints [i.e. external to the vegetation itself] which limit the rate of dry matter production of all or part of the vegetation”. Thus, a gradient of increasing stress is a gradient of decreasing net primary production. Disturbance [2] is “the partial or total destruction of the plant biomass and arises from the activities of herbivores, pathogens, man…, and from phenomena such as wind-damage, frosting, droughting, soil erosion, and fire”. Competition is defined [2] as “the tendency of neighouring plants to utilise the same quantum of light, ion of mineral nutrient, molecule of water, or volume of space”. Therefore, the “phenomena which restrict photosynthetic production” and phenomena that cause the “partial or total destruction of plant biomass” are properties of a habitat that exist in the absence of competing plants. A reduction in the growth of a plant, or even its death, due to resources being captured by another plant is neither a stress nor a disturbance in CSR theory. Within the four permutations of the extremes of stress and disturbance, one (high stress and high disturbance) is untenable because a very high rate of biomass destruction coupled with a very low rate of biomass production prevents any permanent formation of vegetation. The three remaining combinations are associated with the evolution of different suites of correlated traits conforming to each of the three distinct habitat extremes. These are the *C**ompetitors* (low stress and low disturbance), the *S**tress-tolerators* (high-stress and low disturbance) and the *R**uderals* (low stress and high disturbance), thus “CSR” [4]. CSR theory claims that the dynamics and structure of vegetation is a consequence of specific adaptive trade-offs among multiple correlated functional traits (“plant strategies”) with respect to stress and disturbance, such that increased fitness in one circumstance inescapably involves a reduced fitness in another.

The triangular CSR ordination of plants links these theoretical claims with empirical observations. CSR ordination is a practical method of classifying plants and plant communities via functions of various plant functional traits. The empirical CSR ordination method has changed over time. The original CSR ordination [1] used only four traits: canopy height, lateral spread, litter accumulation and maximum relative growth rate in the seeding phase. Species were ordinated into the *C-*dimension according to a competition index, which was a composite of canopy height, lateral spread and litter accumulation. Species were ordinated to the *S-*dimension as a function of maximum relative growth rate (RGR_max_) and ordination of the *R-*dimension was determined by the requirement that the sum of the three dimensions must be 100%. This first ordination method was not practical because RGR_max_ requires growing each species in controlled optimal conditions. A second version of the CSR ordination was proposed for British herbaceous species and was subsequently used as a “gold standard” for all subsequent ordinations that attempt to generalize the CSR scheme; i.e. subsequent versions were constrained to maximally agree with the CSR scores of the species ordinated in this study. The “gold standard” for the *C-*dimension was based on the dominance (relative abundance) of each species in fertile and undisturbed habitats, with greater dominance in fertile, undisturbed habitats conferring a higher *C*-value. The “gold standard” for the *R-*dimension was based on the mean relative abundance of monocarpic species (annuals + biennials + geophytes) in quadrats in which the particular species was present. The “gold standard” for the *S-*dimension was determined by the loadings and scores of the first axis of the PCA of 67 traits on 43 British herbaceous species [5], which was interpreted as representing an axis of resource acquisition *vs*. retention.

In order to generalize these “gold standard” CSR values to new species, without having to obtain extensive field observations and while using fewer and more easily measured traits, [6] proposed a third version of the CSR ordination by regressing seven morphological and phenological traits on the “gold-standard” CSR values of the same 43 British herbaceous species. Using these regression equations and a series of statistical manipulations, one can ordinate any herbaceous species having these seven traits onto the CSR triangle. These seven traits (canopy height, lateral spread, leaf dry weight, leaf dry matter content, specific leaf area, flowering period and flowering starting point) were those, among 67 traits considered, that best predicted the “gold standard” CSR scores. However, this ordination method still had two weaknesses: (i) it still required phenological traits (flowering period and flowering starting point) that are difficult to obtain and to generalize outside of northwestern Europe, and (ii) it could not be extended to vascular plants other than terrestrial herbaceous species. As a result, although Grime’s CSR theory is designed to maximize generality, its empirical application was been mostly limited to British herbs.

To overcome these limitations Pierce et al. [7] proposed a much simpler and more general CSR ordination method by using only three representative, and easily measured, leaf traits: leaf area (representing the plant size spectrum), plus leaf dry matter content and specific leaf area (representing conservative vs. acquisitive resource economics). Diaz et al. [8] have recently validated these two major axes of trait variation using a worldwide trait database. The Pierce et al. [7] method was subsequently extended to thousands of plants worldwide, producing a globally calibrated CSR ordination tool: “StrateFy” [9]. The use of these three leaf traits does not mean that these are the only important traits, or even the most important traits from a functional perspective; rather, they are able to approximately capture the principal multivariate axes of trait variation while being both easy to measure and measurable on any species having leaves. This allows for worldwide comparisons of species and communities based on plant ecological strategies. However, has StrateFy sacrificed too much precision in order to maximize generality and simplicity? How well does this globally calibrated method work in a local area? One objective of our study was to determine the degree to which StrateFy, in conjunction with CSR theory, can correctly predict changes in plant community structure along experimentally manipulated gradients of stress and disturbance in a local study.

CSR ordination also provides an empirical link between CSR theory and the functional structure of natural vegetation, as opposed to individual species, via the concept of community-weighted mean (CWM) CSR values. CWM CSR values of a community estimate the CSR value of an average individual in that community by weighting the CSR values of each species by their relative abundance within the community. For instance, Grime’s original [1] publication used community-weighted trait values (without using the term) to place vegetation plots onto the CSR triangle. Importantly, using CWM CSR scores, one can derive three empirically falsifiable predictions of CSR theory:

1. Increasing levels of environmental stress (i.e. environmental conditions that decrease net primary productivity) will result in vegetation increasingly dominated by species with S-selected traits (thus increasing S_CWM_ values);
2. increasing levels of environmental disturbance (i.e. environmental conditions that increase the amount of plant biomass destruction) will result in vegetation increasingly dominated by species with R-selected traits (thus increasing R_CWM_ values); and
3. decreasing levels of both stress and disturbance will result in vegetation increasingly dominated by species with C-selected traits (thus increasing C_CWM_ values.

How well supported are these predictions? All observational field tests of CSR theory except Eler et al. [10] have subjectively specified the levels of stress and disturbance based on the vegetation itself or else used indirect environmental variables whose relationship to plant productivity (stress) and biomass destruction (disturbance) was not explicitly demonstrated [11–13]. Such studies are not strong tests of CSR theory. A more convincing way of testing CSR theory is to experimentally manipulate levels of stress and disturbance so that these two environmental properties are known and controlled independently of the vegetation. There is, to our knowledge, only one such experimental test with controlled levels of stress and disturbance [14]. This study grew seven contrasting species of grasses in both monocultures and in mixture along experimentally controlled levels of nutrient supply and biomass destruction and compared their responses relative to the predictions by CSR theory. Such an important theory cannot be properly judged by only one experimental study, involving only seven taxonomically restricted species and spanning only two growing seasons from planting to final harvest.

In this study, we report the results of a robust experimental test of CSR theory involving 37 herbaceous species of both grasses and herbs growing in mixture over a five-year period in 24 mesocosms arranged along experimentally maintained gradients of stress and disturbance. Values of C_CWM_, S_CWM_ and R_CWM_ were for each mesocosm using StrateFy in order to test the predicted changes in the community-weighted CSR scores as described above. Furthermore, since the three traits used in the StrateFy ordination were chosen to summarize the responses of a much larger suite of traits implicated in the CSR strategy scheme, we also measured 13 other functional traits and compared their community-weighted mean patterns in relation the CSR theory.

## Materials and methods

### Experimental design

We constructed 24 mesocosms (112.5cm × 90cm × 36cm) at the Université de Sherbrooke, Quebec, Canada (N45.24, W71.54) in 2009. These mesocosms were made of high-intensity plastic, drained from a single point at the bottom, and were maintained outdoors within a10m × 6m area from the beginning of the experiment. Three levels of stress and four levels of disturbance, with two replicates of each combination, were randomly assigned to the 24 mesocosms. The stress gradient, which includes both potential soil nutrient supply and soil water holding capacity (both of which determine actual soil nutrient supply) was obtained by mixing different ratios of garden soil and sand, and a fixed proportion (1/3) of clay soil. The ratio of garden soil to sand was 3:1, 2:2 and 1:3 in low stress (SL), medium stress (SM) and high stress (SH) mesocosms, respectively.

Seeds of 30 herbaceous species (S1 Table), chosen to span a wide range of habitats and functional traits, were mixed in a proportion determined by their germination rates such that each species would have the same initial seedling density. The mixture of seeds was split into two equal parts and each broadcast in opposing directions over the soil surface of each mesocosm to increase seedling uniformity. Over the five years of the experiment, 12 of the originally seeded species disappeared while 19 new species appeared. Most of these new species came from seeds already present in the clay soil (based on germination tests conducted at the start of the experiment) and, since the same amount of this clay soil was added to each mesocosm, the same average number of seeds of each of these new species was also added to each mesocosm at the start of the experiment. However, it is possible that some of the new species arrived from other dispersal vectors.

Each mesocosm was conceptually (not physically) divided into 80 cells of 10 cm × 10 cm and with a 10 cm wide boundary at the edge of each mesocosm as a buffer. The disturbance treatments were applied to a cell by: (i) cutting the vegetation to ground level with the cut biomass being distributed throughout the mesocosm (simulating grazing), (ii) cutting the soil to a depth of 10 cm along the edge of the cell to sever any shallow rhizomes, and (iii) lightly raking the soil surface (simulating activities of small mammals or light agricultural activity). This disturbance was applied annually on 4 or 27 randomly selected cells per mesocosms (i.e. intensity); this was done for each intensity level either once (at the beginning May) or twice (at the beginning of May and at the end of July) each year (i.e. frequency). We did this because Grime’s definition of “disturbance” includes both the intensity and frequency of biomass destruction. Therefore, we obtained four levels of disturbance: D04, D08, D27 and D54, indicating a yearly average disturbance intensity of 4 (5%), 8 (10%), 27 (34%) and 54 (68%) out of 80 cells per mesocosm.

### Species survey and trait measurements

Species abundance (the number of rooted stems per species) was recorded in each of 30 randomly sampled cells per mesocosm just prior to the second disturbance in July 2014 (the 5^th^ year of the experiment). We recorded 37 species from the 24 mesocosms, which varied from 9 to 17 species per mesocosm, in July 2014. During July and August in 2014 we measured the three leaf traits used for the StrateFy [9] CSR ordination: leaf area (LA), leaf dry matter content (LDMC), specific leaf area (SLA). Each trait was measured on five individuals per species per mesocosm according a standardized protocol [15]. Each individual plant was cut at ground level and stored in a cooler with the stem base in water. The cooler was then stored in the dark for a whole night (around 15h) to allow the leaves completely rehydrate and burn off accumulated non-structural carbohydrates so that dry mass represented structural components. One mature and healthy leaf was collected from each individual the next day to measure the traits. We also measured 13 other functional traits (Table 1) related to plant CSR strategy using the Perez-Harguindeguy et al. [16] protocols on these 37 species growing alone in pots. The soil in each pot consisted of same ratio of garden soil and sand as in the medium stress mesocosms and the pots were placed next to the mesocosms. These traits were measured during July and August in 2015 after one growing season except that seed mass and seed sphericity were measured before seeding out.

**Table 1:** Measured traits, their measurement units and definitions.

Trait	Code	Units	Notes
Whole plant traits			
Life history	LH	-	1 for annuals and 0 for perennials
Total biomass	TB	mg	biomass including above- and below-ground biomass
Vegetative height	VH	mm	distance between top photosynthetic tissue and ground level
Leaf traits			
Leaf area	LA	mm^2^	by scanner and ImageJ
Leaf dry matter content	LDMC	%	leaf fresh mass / leaf dry mass
Specific leaf area	SLA	mm^2^ mg^-1^	leaf area / leaf dry mass
Leaf thickness	LT	mm	by micrometer
Leaf carbon concentration	LCC	%	by Elementar analyzer with sample size around 100 mg
Leaf nitrogen concentration	LNC	%	by Elementar analyzer with sample size around 100 mg
Maximum photosynthetic rate	MPR	μmol CO_2_ m^-2^ s^-1^	by Licor 6400 with controlled light intensity (800μmol m^-2^ s^-1^) and leaf temperature (23°C), CO_2_ in air: 380 ppm to 400 ppm
Stem traits			
Stem dry matter content	SDMC	%	stem section fresh mass / stem section dry mass
Specific stem density	SSD	mg mm^-3^	stem section fresh mass / stem section volume
Root traits			
Specific root length	SRL	mm g^-1^	fine root section length / fine root section dry mass
Root biomass	RB	mg	below-ground biomass
Seed traits			
Seed mass	SM	mg	average mass of 20 seeds
Seed sphericity	SS	-	standard deviation of the 3-dimensions of the seeds

### Data analyses

First, species’ mean trait values (S2 Table) were calculated for each species and entered into the “StrateFy” spreadsheet [9] which returned the ternary coordinates and tertiary CSR strategies (S2 Table). The CSR triangle was produced using the “ade4” package [17] of R [18]. Using these C-, S-, and R- values of each species (the ternary coordinates), we then calculated community-weighted mean (CWM) CSR values for each mesocosms as:

$$C_{CWM}=\sum_{i=1}^{n}a_{i}C_{i};\;S_{CWM}=\sum_{i=1}^{n}a_{i}S_{i};\;R_{CWM}=\sum_{i=1}^{n}a_{i}R_{i}$$

where *a*_*i*_, *C*_*i*_, *S*_*i*_ and *R*_*i*_ is the relative abundance and *C*, *S and R* values of the *i*^*th*^ species in the mesocosm. Since *C*_*CWM*_, *S*_*CWM*_ and *R*_*CWM*_ are bounded by 0 and 1 and are constrained to sum to unity, we used permutation ANOVA via the package “lmPerm” [19] in R to test whether *C*_*CWM*_, *S*_*CWM*_ and *R*_*CWM*_ varied significantly along stress and disturbance gradients. We also calculated CSR values for each mesocosm based on species’ trait values obtained by individuals only from that mesocosm, which therefore allows for intraspecific trait variation across mesocosms. Then *C*_*CWM*_, *S*_*CWM*_ and *R*_*CWM*_ were calculated in the same way, except that *C*_*i*_, *S*_*i*_ and *R*_*i*_ referred to CSR values based on population mean trait values but not based on species mean trait values.

## Results

**Fig 1:**
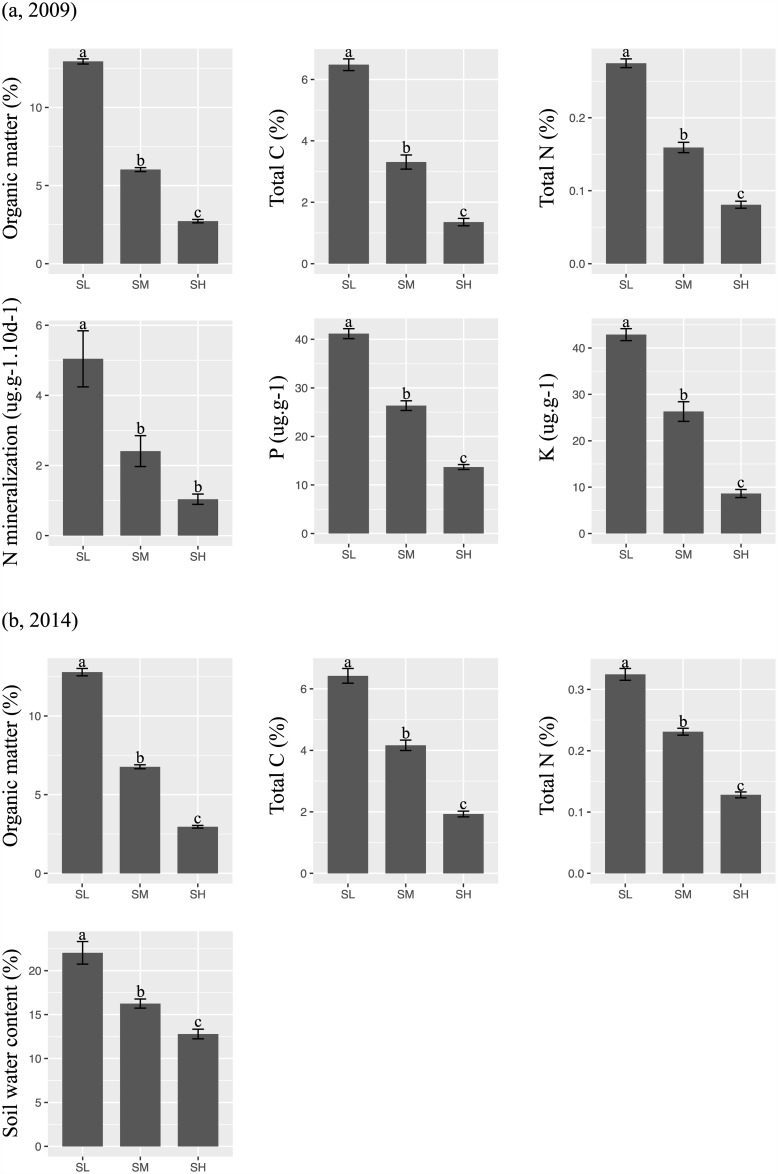
Soil analyses along the stress gradient. Soil analyses (means ±SE) of the mesocosms having different intensities of stress (low stress: SL, medium stress: SM, and high stress: SH) in 2009 (a) and 2014 (b). Letters indicate significant differences in means. Note that decreasing stress implies increasing soil fertility.

**Fig 2:**
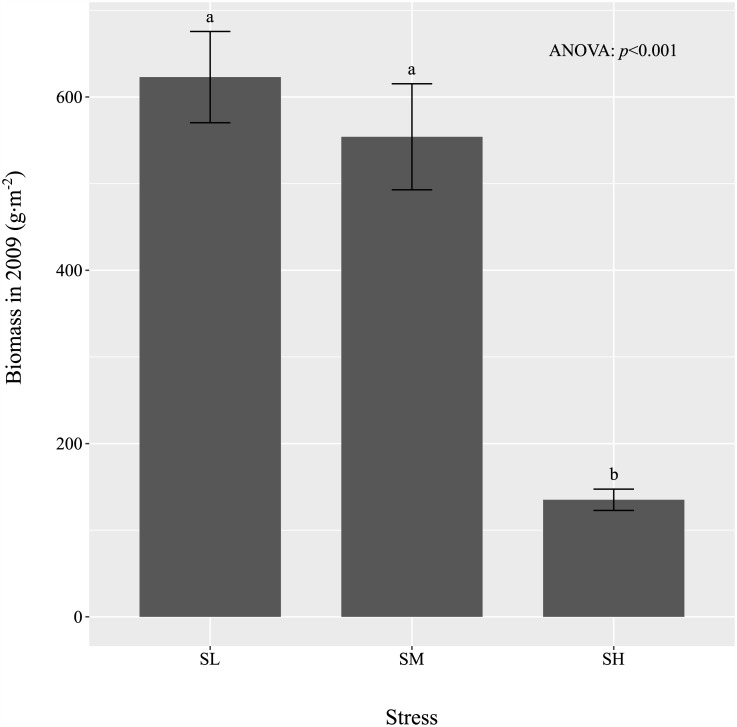
Maximum aboveground biomass along the stress gradient in 2009. Peak aboveground biomass (means ±SE) in 2009 for the 24 mesocosms under different intensity of stress (SL: low stress, SM: medium stress, and SH: high stress). Letters indicate significant differences in means.

Soil fertility levels (organic matter, total C, N, P and K as well as N mineralization and soil volumetric water content) in the high (SH) and medium (SM) stress treatments were approximately 25% and 50% as in the low (SL) treatment (Fig 1a), and this gradient was maintained in 2014 (Fig 1b). These differences in soil resource levels translated into non-linear differences in biomass production; aboveground biomass, collected at the period of peak standing crop during the first year of the experiment (2009) and before any disturbance treatments had been applied, are shown in Fig 2. The high stress treatment produced only 20% of the biomass of the low stress treatment (p<0.001) but the medium stress treatment produced 88% of the biomass of the low stress treatment, and this modest decrease was not significantly different based on a Tukey post-hoc test.

**Fig 3:**
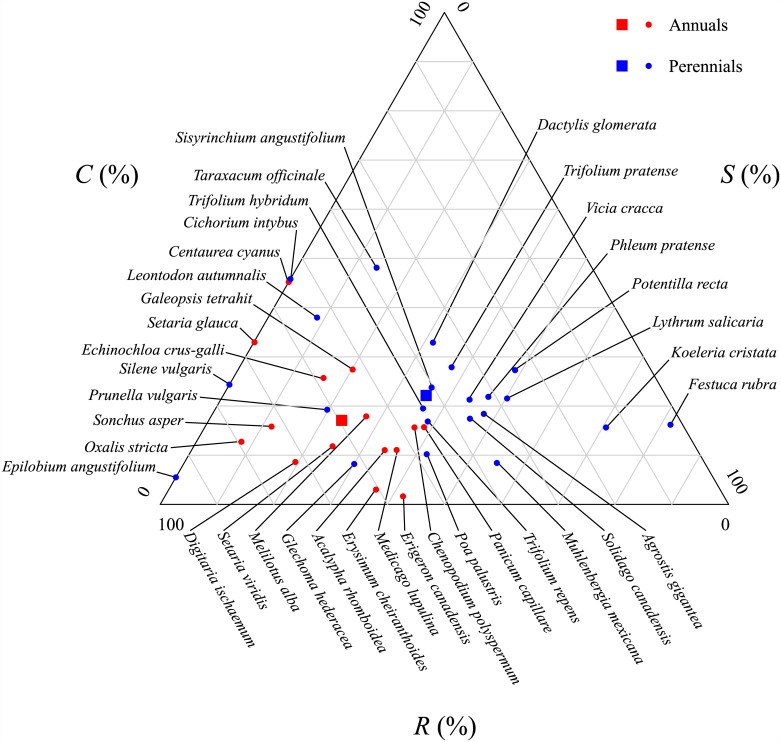
CSR ordination of individual species. Ordination of the 37 species to the CSR triangle using StrateFy. Red dots represent annual (A) species while blue dots represent perennial (P) species. Red and blue squares indicate the average CSR values for annuals and perennials, respectively.

The triangular CSR plot (Fig 3) shows the ordination of the 37 species. The S2 Table lists the trait values and the resulting C, S and R scores for each species. No species occupied the *C-*corner of the triangle, while the *S-*corner of the triangle included slow-growing perennials such as *Festuca rubra* L., and *R-*corner of the triangle consisted of fast-growing annuals such as *Digitaria ischaemum* (Schreb.) Muhl. On average, annual species had significantly higher *R*-values (p<0.001) and lower *S*-values (p = 0.044) than the perennial species and no significant difference of *C*-values (p = 0.119) was found between the two life-history types (Fig 3).

**Fig 4:**
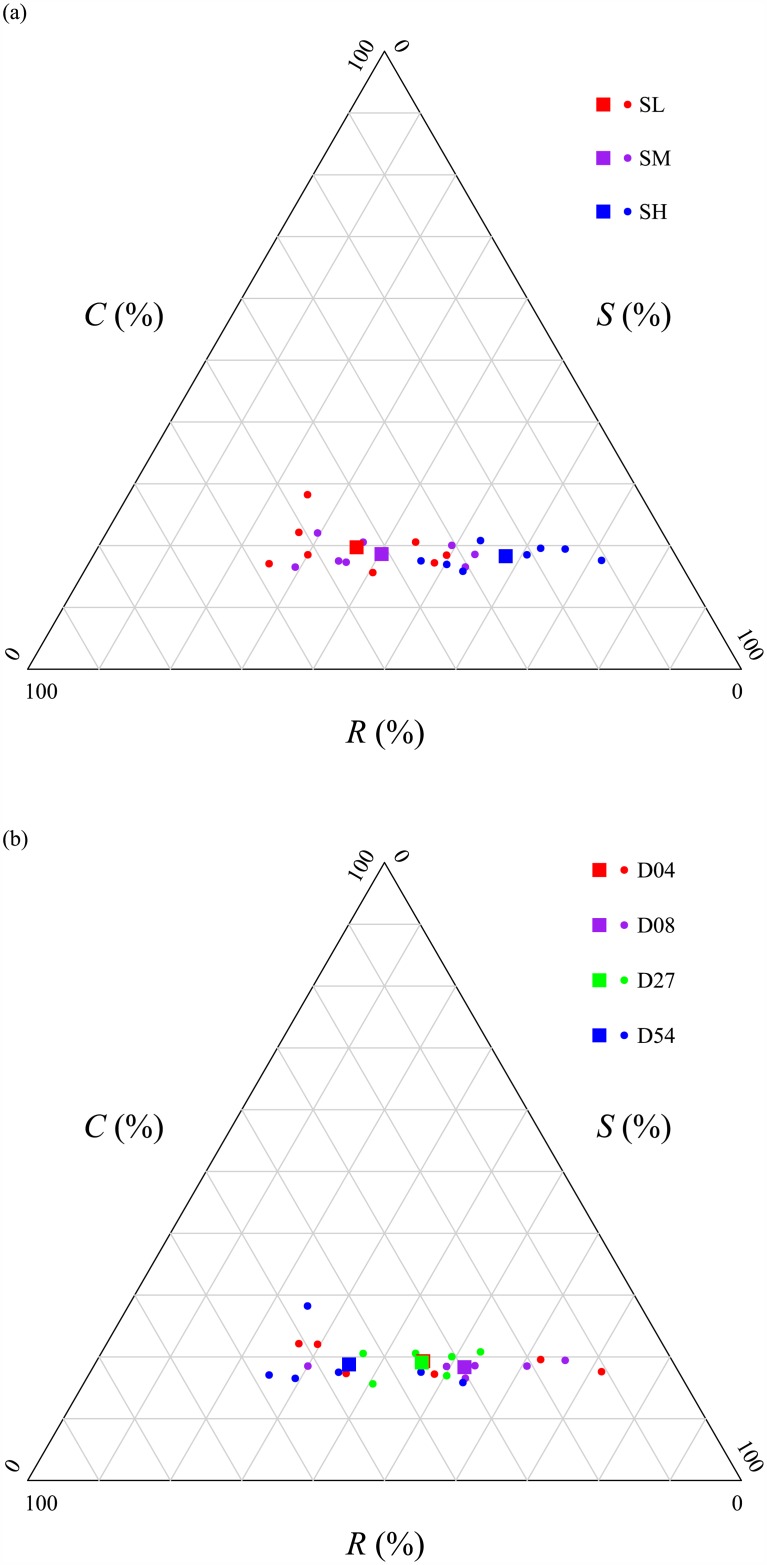
CSR ordination of community-weighted values. Community-weighted mean CSR values (*C*_*CWM*_, *S*_*CWM*_ and *R*_*CWM*_) of the 24 mesocosms under different intensities of stress (a) and disturbance (b). In (a), dots in red, purple and blue represent mesocosms under low stress (SL), medium stress (SM) and high stress (SH), respectively. Squares in red, purple and blue indicate the average CSR values for SL, SM and SH, respectively. In (b), dots in red, purple, green and blue represent mesocosms under different levels of disturbance: D04, D08, D027 and D54 (4, 8, 27 and 54 out of 80 cells disturbed per mesocosm each year). Squares in red, purple, green and blue indicate the average CSR values for D04, D08, D027 and D54, respectively.

The next triangular CSR plot (Fig 4) shows the ordination of the vegetation in the 24 mesocosms based on the community-weighted C, S and R scored. *S*_*CWM*_ and *R*_*CWM*_ each differed significantly between the different levels of stress and disturbance but without any interaction (Table 2), while *C*_CWM_ did not differ significantly between different levels of stress and disturbance (Table 2), either alone or in interaction. The S3 Table lists the trait values and the resulting community-weighted C, S and R scores for each mesocosm. Specifically, *S*_*CWM*_ was higher and *R*_*CWM*_ was lower in high stress (SH) mesocosms than in medium and low stress (SM and SL) mesocosms (Fig 4a), *R*_*CWM*_ was higher and *S*_*CWM*_ was lower in most disturbed (D54) mesocosms than in other intensity of disturbance (D27, D08 and D04) mesocosms (Fig 4b). The results obtained when including the intraspecific trait variability were almost identical to the ordination by using species’ mean trait values.

**Table 2:** Permutation ANOVAs of community-weighted mean CSR values (*C*_CWM_, *S*_CWM_ and *R*_CWM_) along gradients of stress and disturbance.

Response	Source	DF	SS	MS	p
C_CWM_	Stress	2	9.086	4.543	0.671
	Disturbance	3	3.203	1.068	0.969
	interaction	6	44.431	7.405	0.602
	Residuals	12	111.764	9.314	
S_CWM_	Stress	2	2111.608	1055.804	<0.001
	Disturbance	3	827.608	275.869	0.017
	interaction	6	545.538	90.923	0.198
	Residuals	12	625.980	52.165	
R_CWM_	Stress	2	1897.366	948.683	<0.001
	Disturbance	3	801.283	267.094	0.016
	interaction	6	480.671	80.112	0.233
	Residuals	12	600.782	50.065	

Shown are the degrees of freedom (DF), the sum of squares (SS) and mean square (MS) of the analysis of variance. The resulting permutation null probabilities (p) are based on 999999 independent permutation iterations.

Table 3 shows the Pearson correlation coefficients between the community-weighted means of the 16 traits and the three community-weighted means of the CSR values. Of the 48 correlation coefficients, 21 (44%) were significant at the 5% level. Of these 21 significant correlations, only two were in opposite directions to that expected from CSR theory: maximum net photosynthetic rate and specific root length were each expected to decrease with increasing scores on the stress tolerator axis but instead they were positively correlated. Of the 16 traits, the community-weighted values of eight of them varied significantly along at least one of the two experimental treatments (Table 4).

**Table 3:** Observed Pearson correlations between community-weighted mean (CWM) trait values and CWM CSR values (*C*_CWM_, *S*_CWM_, *R*_CWM_).

Traits_CWM_	C_CWM_	S_CWM_	R_CWM_
LH	-0.25 (-)	**-0.74** (-)	**0.82** (+)
TB	0.26 (+)	-0.03 (-)	-0.02 (-)
VH	-0.04 (+)	0.25 (-)	-0.25 (-)
LA	**0.87** (+)	**-0.43** (-)	0.26 (-)
LDMC	-0.29 (-)	**0.93** (+)	**-0.91** (-)
SLA	-0.1 (+)	**-0.85** (-)	**0.91** (-)
LT	-0.13 (?)	**0.9** (+)	**-0.91** (?)
LCC	-0.12 (?)	-0.35 (+)	0.39 (-)
LNC	-0.04 (+)	**-0.78** (-)	**0.82** (+)
MPR	-0.11 (+)	**0.54** (-)	**-0.54** (+)
SDMC	-0.13 (+)	0.2 (+)	-0.18 (-)
SSD	0.13 (+)	-0.14 (+)	0.11 (-)
RB	**0.58** (+)	0.35 (-)	**-0.49** (-)
SRL	**-0.51** (+)	**0.36** (-)	-0.27 (+)
SM	-0.11 (+)	-0.14 (+)	0.17 (-)
SS	**0.52** (+)	**0.45** (?)	**-0.58** (-)

Correlations in bold are significant (p<0.05), symbols in brackets indicate the signs of the correlations predicted by CSR theory as positive (+), negative (-) or unclear (?). Trait abbreviations as in Table 1.

**Table 4:** Permutation ANOVAs of community-weighted trait means along gradients of stress and disturbance.

Trait	Source	DF	SS	MS	P
LH	Stress	2	0.239	0.119	0.081
	Disturbance	3	0.142	0.047	0.337
	interaction	6	0.303	0.050	0.316
	Residuals	12	0.454	0.038	
TB	Stress	2	26820.033	13410.017	0.373
	Disturbance	3	69201.442	23067.147	0.197
	interaction	6	168844.178	28140.696	0.116
	Residuals	12	152061.767	12671.814	
VH	Stress	2	7474.114	3737.057	0.392
	Disturbance	3	33887.626	11295.875	0.066
	interaction	6	33518.371	5586.395	0.251
	Residuals	12	44154.884	3679.574	
LA	Stress	2	93835.750	46917.875	0.201
	Disturbance	3	40787.848	13595.949	0.673
	interaction	6	83552.421	13925.404	0.767
	Residuals	12	306265.954	25522.163	
LDMC	Stress	2	48.121	24.061	0.032
	Disturbance	3	40.418	13.473	0.100
	interaction	6	28.375	4.729	0.508
	Residuals	12	61.393	5.116	
SLA	Stress	2	479.731	239.866	0.001
	Disturbance	3	442.812	147.604	0.005
	interaction	6	142.413	23.736	0.406
	Residuals	12	254.419	21.202	
LT	Stress	2	0.124	0.062	0.000
	Disturbance	3	0.029	0.010	0.001
	interaction	6	0.022	0.004	0.009
	Residuals	12	0.008	0.001	
LCC	Stress	2	0.990	0.495	0.191
	Disturbance	3	0.033	0.011	0.987
	interaction	6	0.696	0.116	0.830
	Residuals	12	3.120	0.260	
LNC	Stress	2	2.147	1.073	0.005
	Disturbance	3	0.179	0.060	0.726
	interaction	6	0.968	0.161	0.357
	Residuals	12	1.581	0.132	
MPR	Stress	2	7.541	3.770	0.194
	Disturbance	3	16.998	5.666	0.084
	interaction	6	27.668	4.611	0.102
	Residuals	12	23.815	1.985	
SDMC	Stress	2	11.468	5.734	0.276
	Disturbance	3	60.825	20.275	0.018
	interaction	6	61.999	10.333	0.077
	Residuals	12	47.656	3.971	
SSD	Stress	2	0.007	0.004	0.024
	Disturbance	3	0.007	0.002	0.053
	interaction	6	0.011	0.002	0.067
	Residuals	12	0.008	0.001	
RB	Stress	2	2972.163	1486.081	0.466
	Disturbance	3	6020.156	2006.719	0.390
	interaction	6	14794.393	2465.732	0.321
	Residuals	12	22295.912	1857.993	
SRL	Stress	2	5546.527	2773.264	0.003
	Disturbance	3	1665.090	555.030	0.182
	interaction	6	5357.724	892.954	0.047
	Residuals	12	3493.822	291.152	
SM	Stress	2	35.610	17.805	0.008
	Disturbance	3	5.792	1.931	0.491
	interaction	6	24.233	4.039	0.179
	Residuals	12	26.835	2.236	
SS	Stress	2	0.365	0.182	0.067
	Disturbance	3	0.085	0.028	0.667
	interaction	6	0.153	0.026	0.807
	Residuals	12	0.638	0.053	

Shown are the degrees of freedom (DF), the sum of squares (SS) and mean square (MS) of the analysis of variance. The resulting permutation null probabilities (p) are based on 999999 independent permutations.

## Discussion

A test of CSR theory that is not logically circular requires that one be able to specify the levels of stress and disturbance experienced by a site independently of the functional signature of the vegetation itself. This can be done by measuring the rates of net primary production or determinants of this (decreasing values being more stressful by definition) and of live biomass destruction (increasing values being more disturbed by definition). Because of the practical difficulties involved in obtaining these environmental measurements, this has seldom been done in practice for field studies of plant strategies (but see [10]). Instead, sites in these field studies were qualitatively classified in terms of stress and disturbance based on vegetation structure [11–13], which leads a degree of circularity. For instance, Grime et al.’s [12] comprehensive work only classified habitats as “wetland”, “skeletal”, “arable” etc. A strength of our experimental design is that the levels of soil resource supply (stress) and the intensity of biomass destruction (disturbance) were measured independently of the functional structure of the vegetation and fixed at different levels for the duration of the experiment. Although it is impossible to include all possible natural causes of biomass destruction in an experiment, we believe that our causes of disturbance (clipping to simulate herbivore grazing and superficial soil raking to simulate the activities of small mammals) are reasonable and mirror those used in the experiment of Campbell and Grime [14]. Our soil nutrient measurements showed that our three “stress” levels corresponded to three levels of soil nutrient and water availability. Aboveground biomass, collected during the application of the disturbance in the first year, showed that increased soil resource availabilities along stress gradient did translate into increased biomass production. However, at least based on the first year results, the linear increase in soil nutrients over the three stress treatments did not translate into a linear increase in plant biomass production, since the “medium” and “low” stress treatments did not differ statistically in terms of biomass production. Therefore, this allows us to test CSR theory with the StrateFy ordination in a non-circular manner.

The global StrateFy CSR ordination correctly predicted the expected responses to stress and disturbance.

CSR theory predicts that an increase in stress (i.e. a decrease in soil fertility) will cause the plant community to shift towards one more dominated by stress tolerators while an increase in disturbance will cause the plant community to shift towards one more dominated by ruderals. The *StrateFy* method, if correct, must mirror this. Our results (Fig 2) confirm both of these predictions. Furthermore, the *S*_*CWM*_ and *R*_*CWM*_ values were significantly correlated to CWM values of many other traits as predicted by CSR theory (Table 3). Therefore, the *S-* and *R-*dimensions identified by StrateFy with only three leaf traits (leaf surface area, leaf dry matter content and specific leaf area) correctly captured information from other traits and correctly predicted how most of the other CWM trait values would change as a function of stress and disturbance as specified by CSR theory. For instance, increasing community-weighted values along the S dimension were significantly associated with vegetation having fewer annuals (r = -0.74), thicker leaves (r = 0.90), less leaf nitrogen per mass (-0.78), higher specific root lengths (r = 0.36), and less spherical seeds (r = 0.45). However, contrary to expectations, S_CWM_ was also associated with higher maximum net photosynthetic rates and a higher specific root length (i.e. thinner or less dense root systems). Increasing community-weighted values along the R dimension were associated with more annuals (r = 0.82), thinner leaves (r = -0.91), more leaf nitrogen per mass (r = 0.82), smaller root systems (r = -0.49) and more spherical seeds (r = -0.58).

The global StrateFy CSR ordination did not predict the expected responses to competition.

CSR theory predicts that competitors will dominate in mesocosms having low stress and low disturbance (i.e. fertile conditions with little biomass destruction). Contrary to this expectation, our measures of *C*_*CWM*_ did not significantly decline as the intensity of stress or disturbance increased. This contrasts with the only other experimental test of CSR theory [14] which did find that absolute reductions in biomass and flowering due to competition were greatest at low stress and low disturbance. Species classified as stress tolerators or ruderals in that study experienced this competitive effect most strongly in agreement with the predictions by CSR theory. Here, we consider three different hypotheses to explain our results.

One possible reason why our *C*_*CWM*_ did not behave as expected is that the *C-*dimension was not properly identified by StateFy, which uses only three leaf traits. In StrateFy, high values along the C-dimension occur when a species has large leaves (leaf area) but intermediate values of SLA and LDMC (the economic axis). The reason for using only leaf area in StrateFy, but not traits like vegetative height, is to allow comparisons between widely different growth forms and habitats such as herbaceous and woody species or aquatics. However, the gain in generality obtained by using only three easily measured leaf traits might have resulted in too much of a loss in precision with respect to the hypothesized link to competitive ability. Perhaps no species in our study occupied the *C-*corner of the CSR triangle because of an underestimation of the *C-*dimension using the global StrateFy ordination? We don’t think that this explanation is correct. Independently of the StrateFy ordination, none of the CWM trait values in our data that were related to plant size (total biomass, vegetative height, leaf area) varied significantly along our gradients of stress and disturbance (Table 4). In other words, we would have obtained similar results even if we had used other size-related traits to estimate the *C*-dimension. It therefore seems unlikely that the StrateFy ordination itself caused this result.

A second possible explanation for the absence of a response along the C_CWM_ dimension, and the absence of species in the *C*-corner of the CSR triangle, might be that the experimental intensities of stress and disturbance imposed on our mesocosms were not sufficiently low to select for strong competitors. In other words, perhaps the levels of soil nutrients provided in our “low” stress (i.e. high fertility) treatment were not sufficiently high. We don’t think this is the case. The aboveground biomass did increase as stress decreased, but the increase was not significant between medium stress and low stress (i.e. between medium and high fertility) even though the soil nutrient levels did increase significantly (Fig 1) between the medium and low stress mesocosms. This suggests that the soil nutrient availabilities in our low stress (i.e. high fertility) mesocosms exceeded the requirements of the plants in our species pool and so did not result in significantly higher aboveground biomass production. If so then this should be sufficient to select for more competitive species in our lowest disturbance levels (5% of area disturbed per year).

Therefore, we think that the most likely explanation for our lack of response in *C*_CWM_, and an absence of species in the *C*-corner, is that we did not include sufficiently strong competitors in our species pool to be selectively favoured in the low stress, low disturbance mesocosms. For instance, herbaceous plants classified as highest on the C axis by Grime et al. [12] have vegetative heights of 1.5m or more (for example *Epilobium hirsutum* L., *Typha latifolia* L., *Epilobium hirsutum* L. or *Urtica doiica* L.), while the tallest of our species in the year of measurement (i.e. *Lythrum salicaria* L., *Trifolium hybridum* L. and *Phleum pratense* L.) had average vegetative heights around 0.6m (S2 Table). It is important to remember that the StrateFy ordination is calibrated to cover a worldwide range of plant traits and resulting CSR scores, including trees. Thus, the most likely explanation for our result is that the range of trait variation in relation to the C dimension that was exhibited by our species was simply too narrow to be detected given the levels of replication available in such an experimental design.

## Conclusions

Our results are the first experimental evidence that CSR theory can (mostly) predict the variation in functional traits during community assembly of herbaceous vegetation along gradients of net primary productivity (stress) and density-independent mortality (disturbance). Furthermore, we show that these multivariate patterns of functional traits (“ecological strategies”) can be captured in a local study using only three easily measured leaf traits using StrateFy even though StrateFy is calibrated using the global range of trait variation for these traits. This is important because these three leaf traits are particularly well represented in the TRY trait database [20]. In combination with large vegetation databases of community taxonomic composition and abundance, it should be possible to infer both the CSR vegetation structure and changes in such structure as a consequence of manipulations in soil fertility and disturbance regimes associated with land use. However, before this can be done it will be necessary to develop methods of quantitatively inferring net primary productivity (thus stress) and plant biomass destruction (disturbance) from environmental variables and then quantitatively linking these to the CSR signature of the vegetation [21]

## Supporting information

S1 Table List of species included in the experiment. (DOCX)

S2 Table Species’ mean trait values and CSR values. See abbreviations and units of traits in Table 1 of the main paper.
(XLSX)

S3 Table Community-weighted mean (CWM) trait and CWM CSR values. See abbreviations and units of traits in Table 1 of the main paper.
(XLSX)
