# Supplementary material for: An experimental test of CSR theory using a globally calibrated ordination method
Source: PLoS One. 2017 Apr 7;12(4):e0175404. doi: 10.1371/journal.pone.0175404 (PMC5384788; doi:10.1371/journal.pone.0175404)
Supplement: S1 Table — (DOCX) [file pone.0175404.s001.docx]

**Table S1. List of species.**

| Original species planted in 2009 | Species survey in 2014  014 presenturveyed in 2014 |
| --- | --- |
| **Still present in 2014** | **Original species planted in 2009** |
| *Agrostis gigantea* Roth. | *Agrostis gigantea* Roth. |
| *Centaurea cyanus* Hill. | *Centaurea cyanus* Hill. |
| *Chenopodium polyspermum* L. | *Chenopodium polyspermum* L. |
| *Cichorium intybus* L. | *Cichorium intybus* L. |
| *Dactylis glomerata* L. | *Dactylis glomerata* L. |
| *Epilobium angustifolium* (L.) Holub | *Epilobium angustifolium* (L.) Holub |
| *Erigeron canadensis* L. | *Erigeron canadensis* L. |
| *Festuca rubra* L. | *Festuca rubra* L. |
| *Koeleria cristata* (Ledeb.) Schult. | *Koeleria cristata* (Ledeb.) Schult. |
| *Leontodon autumnalis* Oed. | *Leontodon autumnalis* Oed. |
| *Lythrum salicaria* L. | *Lythrum salicaria* L. |
| *Phleum pratense* L. | *Phleum pratense* L. |
| *Poa palustris* L. | *Poa palustris* L. |
| *Potentilla recta* L. | *Potentilla recta* L. |
| *Prunella vulgaris* L. | *Prunella vulgaris* L. |
| *Silene vulgaris* Garcke. | *Silene vulgaris* Garcke. |
| *Solidago canadensis* L. | *Solidago canadensis* L. |
| *Trifolium pratense* L. | *Trifolium pratense* L. |
| **No longer present in 2014** | **Naturally invaded species since 2009** |
| *Althaea officinalis* L. | *Acalypha rhomboidea* Raf. |
| *Artemisia absinthium* L. | *Digitaria ischaemum* (Schreb.) Muhl. |
| *Beckmannia syzigachne* (Steud.) Fern. | *Echinochloa crus-galli* (L.) Beauv. |
| *Cerastium tomentosum* L. | *Erysimum cheiranthoides* L. |
| *Echium vulgare* L. | *Galeopsis tetrahit* L. |
| *Hyoscyamus niger* L. | *Glechoma hederacea* L. |
| *Oenothera biennis* L. | *Medicago lupulina* L. |
| *Rudbeckia hirta* L. | *Melilotus alba* Desr. |
| *Rumex acetosella* L. | *Muhlenbergia mexicana* (L.) Trin. |
| *Rumex obtusifolius* L. | *Oxalis stricta* L. |
| *Sporobolus cryptandrus* (Torr.) A. Gray. | *Panicum capillare* L. |
| *Stellaria media* (L.) Villars. | *Setaria glauca* (L.) Beauv. |
|  | *Setaria viridis* (L.) Beauv. |
|  | *Sisyrinchium angustifolium* Mill. |
|  | *Sonchus asper* (L.) Hill. |
|  | *Taraxacum officinale* Webb. |
|  | *Trifolium hybridum* L. |
|  | *Trifolium repens* L. |
|  | *Vicia cracca* Benth. |

List of species (i) originally planted in 2009 and still present 2014, (ii) originally planted in 2009 but subsequently extinct and (iii) naturally invaded since 2009 and still present in 2014.
